# 4302 Decreased structural basal ganglia motor loop connections in Vascular Parkinsonism compared to Parkinson’s disease and healthy aging

**DOI:** 10.1017/cts.2020.295

**Published:** 2020-07-29

**Authors:** Christine Cooper, Federico Rodriguez-Porcel, Travis Turner, Gonzalo Revuelta, Jens Jensen, Vanessa Hinson, Leonardo Bonilha

**Affiliations:** 1 Medical University of South Carolina

## Abstract

OBJECTIVES/GOALS: This study uses diffusion kurtosis imaging (DKI) to investigate the structural profiles of basal ganglia (BG) motor circuitry in Vascular Parkinsonism (VP), Parkinson’s disease (PD), and healthy aging controls (HC). VP is a clinical diagnosis of lower body predominant parkinsonism without significant benefit from levodopa. VP is distinct from PD, yet the concept of VP remains debated due to the inability of prior studies to identify specific causative changes. One reason for this may be limitations in measuring intricate BG connectivity in vivo. Given the predominant lower body parkinsonism symptoms in VP, we hypothesized that VP would be associated with decreased connectivity specifically within the BG motor loop. METHODS/STUDY POPULATION: We obtained DKI brain imaging in subjects with VP (N = 7), PD (N = 21), and HCs (N = 58), the latter of which had cardiovascular risk factors but no neurological symptoms. The VP and PD groups were evaluated by a parkinsonism-focused motor exam and brief cognitive testing. We compared BG motor loop connectivity between groups and investigated for correlation between connectivity and clinical scores. To account for differences in fiber counts due to the different imaging scanners and protocols between cohorts, we used a BG motor loop proportion, which was the ratio of the BG motor loop fiber count over a control loop, the visual processing pathway. We used Kruskal-Wallis rank sum test with post-hoc Dunn tests to assess imaging findings between subject groups, and Pearson’s correlation to look for correlation between clinical scores and fiber counts. RESULTS/ANTICIPATED RESULTS: The whole brain connectome showed the fewest number of fibers in VP, followed by PD, and then HC (p<0.0001). The BG motor loop proportion fiber count of the BG motor loop was lower in the VP group, compared to the PD and HC cohorts (p = 0.031). In the VP group, the whole brain connectome fiber count correlated with a gait and balance subscore of the Movement Disorders Society - Unified Parkinson Disease Rating Scale (R = −0.87, p = 0.01). DISCUSSION/SIGNIFICANCE OF IMPACT: This study indicates that VP is associated with decreased structural connectivity, with a disproportionate degree of loss in the BG motor circuitry. While the etiology for this susceptibility to injury and preferential damage to BG remains to be defined, these findings can provide an important starting point for a biological understanding of VP, and a potential future marker for diagnosis and tracking disease progression.

